# Genetic Diversity in Natural Populations of *Rhodiola* Species of Different Adaptation Strategies

**DOI:** 10.3390/genes14040794

**Published:** 2023-03-25

**Authors:** Nina V. Terletskaya, Ainur S. Turzhanova, Oxana N. Khapilina, Moldir Z. Zhumagul, Nataliya D. Meduntseva, Nataliya O. Kudrina, Nazym K. Korbozova, Serik A. Kubentayev, Ruslan Kalendar

**Affiliations:** 1 Faculty of Biology and Biotechnology, Al-Farabi Kazakh National University, Al-Farabi Avenue 71, Almaty 050040, Kazakhstan; teni02@mail.ru; 2 Institute of Genetic and Physiology, Al-Farabi Avenue 93, Almaty 050040, Kazakhstan; nat.mdnt@gmail.com (N.D.M.); kudrina_nat@mail.ru (N.O.K.); naz-ik@mail.ru (N.K.K.); 3 National Center for Biotechnology, Qorghalzhyn Hwy 13, Astana 010000, Kazakhstan; turzhanova-ainur@mail.ru (A.S.T.); oksfur@mail.ru (O.N.K.); 4 Astana International University, Kabanbai Batyr 8, Astana 010000, Kazakhstan; mzhakypzhan@mail.ru; 5 Astana Botanical Garden, Orunbur 16, Astana 010000, Kazakhstan; kubserik@mail.ru; 6 National Laboratory Astana, Nazarbayev University, 53 Kabanbay Batyr Ave., Astana 010000, Kazakhstan; 7 Institute of Biotechnology, Helsinki Institute of Life Science (HiLIFE), University of Helsinki, 00014 Helsinki, Finland

**Keywords:** *Rhodiola* species, abiotic stress, sexual systems, polymorphism, superoxide dismutase, auxin response factors, inter-primer binding site amplification profiling

## Abstract

Representatives of the *Crassulaceae* family’s genus *Rhodiola* are succulents, making them distinctive in a changing environment. One of the most significant tools for analyzing plant resources, including numerous genetic processes in wild populations, is the analysis of molecular genetic polymorphism. This work aimed to look at the polymorphisms of allelic variations of the superoxide dismutase (SOD) and auxin response factor (ARF) gene families, as well as the genetic diversity of five *Rhodiola* species, using the retrotransposons-based fingerprinting approach. The multi-locus exon-primed intron-crossing (EPIC-PCR) profiling approach was used to examine allelic variations in the SOD and ARF gene families. We implemented the inter-primer binding site (iPBS) PCR amplification technique for genome profiling, which demonstrated a significant level of polymorphism in the *Rhodiola* samples studied. Natural populations of *Rhodiola* species have a great capacity for adaptation to unfavorable environmental influences. The genetic variety of wild populations of *Rhodiola* species leads to their improved tolerance of opposing environmental circumstances and species evolutionary divergence based on the diversity of reproductive systems.

## 1. Introduction

There is great interest worldwide in the pharmacological compounds of the various *Rhodiola* species [1,2], the extracts of which have been used for centuries in traditional European and Chinese medicine to increase stamina, reduce the effects of ageing, and treat a variety of diseases [3,4,5,6]. With the implementation of the Nagoya Protocol in 2021, the search for new domestically viable medicinal plant resources has become particularly important for each country [7]. Due to habitat decline and overexploitation to satisfy the growing commercial demand, the *Rhodiola rosea* L. is now an endangered species and included in the Red List of protected plant species in many countries throughout the world [8]. As a result, a complete investigation of other members of this family is required, in terms of both their biological and pharmacological activity, as well as the numerous methods of adaptation to abiotic stress factors.

Representatives of the Crassulaceae family’s genus *Rhodiola* have life cycles driven by similar factors: substantial temperature changes during the day, intense solar radiation, summer snowfalls and vice versa, soil denudation in winter, and deep freezing [9]. These species’ capacity to adapt make them of special importance in today’s rapidly changing climate.

Despite the well-established significance of *Rhodiola* species, our understanding of their distribution, valuable secondary metabolites, adaptive mechanisms, and evolutionary relationships is still limited. *Rhodiola* was first distinguished from Sedum by Linnaeus in 1753 due to its dioecious reproductive system. However, further research is needed to fully comprehend the genus and its characteristics [2]. *Rhodiola* species are recognized as forming a single phylogenetic group [8]. At the same time, Ohba classified four *Rhodiola* subgenera of the genus Sedum (*Crassulaceae*) based on flower features (unisexual or bisexual), basal leaves, and inflorescences, i.e., subgenera of *Rhodiola*, *Primuloides, Crassipedes*, and *Clementias*, including *R. semenovii* (Regel and Herder) Boriss [9,10]. *Rhodiola* differs from other subgenera in being dioecious [8]. Clementsia is a hermaphroditic subgenus [11]. Ohba, on the other hand, believed the *Rhodiola algida* to be part of the subgenus *Rhodiola*, which is distinguished by dioecious, unisexual flowers [12], whereas the flowers in representatives of sect. *Algida* are generally hermaphroditic [13]. The convergent development of dioeciousness in *Rhodiola*, on the other hand, shows that dioeciousness should be utilized with caution in the genus’s intrageneric classification [13]. There is speculation that the explanation for the evolution of dioecious *Rhodiola* may lie in abiotic pollination, which leads to increased seed or pollen production and is most functional in high mountain regions [14].

Previously, we considered certain physiological and phytochemical aspects of the adaptation of individual representatives of the *Crassulaceae* family under laboratory conditions of weak and moderate abiotic stresses, such as drought, salinity, and low positive temperatures, which are not lethal for succulents but activate their adaptation mechanisms and stimulate the formation of biologically active secondary antioxidant metabolites [15,16], as well as their adaptive mechanisms in the dynamics of vegetation in situ [17].

The approach related to sexual differentiation in plants is interesting for studying the mechanisms of adaptation to abiotic stressors [18], but currently, little is known about the processes of plant adaptation and responses to environmental stressors in species belonging to the same genus but differing in mode of reproduction [19].

Many features of development and adaptation have phytochemical expression, but all changes in the cell and the entire organism are regulated by the structure and function of the genome, its genes, and epigenetic alterations [20]. Since most indications of stress tolerance in plants are polygenic, it is difficult to interpret them using solely physiological and biochemical techniques. The tolerance of a plant population of abiotic stimuli is primarily determined by the degree of genetic variation in the key genes that govern plant physiological functions [21]. Thus, one of the most significant tools for understanding genetic processes in wild populations is the investigation of molecular genetic polymorphism, which includes hidden genetic variability in both the coding and noncoding parts of the genome. The study of several protein families and isoenzymes involved in numerous metabolic pathways, as well as research into the involvement of phytohormones in the control of growth, blooming, and daily cycles in plants, is of special interest.

Proteins of the superoxide dismutase (SOD) family, which are found in all plant species and are distinguished by tissue specificity and the ability of these genes to be activated in response to abiotic stresses, are recognized to be key antioxidant enzymes. The auxin response factor (ARF) genes play a crucial role in auxin (one of the most significant groups of phytohormones) signaling and are implicated in plant responses to growth, development, and stress [22,23]. The response to abiotic stresses and the activity of ARF genes can be linked in succulent plants of the *Crassulaceae* family [24].

Apart from genes coding for isoenzymes, the noncoding component of the genome is of special importance, where eukaryotes have abundant transposable or mobile elements that can replicate parasitically in the host genome [25,26]. These multiple transposable elements play a vital role in all eukaryotic genomes, including the plant genome, and are the primary source of epigenetic alterations under stressful circumstances, as well as probably being the primary cause of species evolution. Studies of the noncoding part of the genome can reveal particular signs of hidden genetic diversity and, more broadly, indicators of the genetic potential and evolutionary changes happening in a specific species [26,27,28,29]. Mobile elements are important in the distribution of cis-regulatory elements, which contribute to genetic control in both the short term (adaptation to environmental changes) and the long term (evolutionary changes) [26,30,31].

PCR methods for the identification of hidden genetic variation, such as a system of genome-profiling molecular markers, were created based on sequences for multiple families of complex interspersed genomic repeats [32]. These genome-profiling PCR techniques for investigating genetic variation in eukaryotes, using a multicopy and genomic abundance of transposable elements and endogenous viruses, can extend knowledge of phylogenetic relationships and estimate the genetic diversity of particular species [33,34].

Interspersed repeats-based genome-profiling applications offer a simple and cost-effective PCR method to study individual genetic polymorphisms [33,35]. This genome profiling is based on the fact that transposable elements (in particular, long terminal tepeat (LTR) retrotransposons) are distributed throughout the genome and are involved in recombination events that occur during meiosis. PCR with a single primer corresponding to conserved sequences in LTR retrotransposons is one of the most prominent examples of interspersed repeats-based genome-profiling applications. Specifically, PCR methods based on the detection of transposable element insertion site polymorphisms include inter-retrotransposon amplified polymorphism (IRAP) and retrotransposon–microsatellite amplified polymorphism (REMAP) [33]. The inter-primer binding site (iPBS) amplification technique also proved to be a powerful genome fingerprinting method that does not require information about retrotransposon sequences [36]. Furthermore, new enhanced methods for high-throughput targeted gene characterization and transposon display were added to current methodologies, which may be modified to adopt high-throughput sequencing technologies, among other things. For example, palindromic sequence-targeted (PST) PCR utilizes a pair of primers, one of which is complementary to 6 bp long palindromic sequences (PST sites) and the other of which conserves transposable element sequences [37,38]. The PST-PCR technique allows genome walking and profiling that can be used in initial descriptions of intraspecific and interspecies genetic variability and to track lines and genotypes [37]. 

The study of the genetic diversity of the gene families of SOD and ARF in *Rhodiola* species differing in the mode of reproduction in conjunction with genetic analysis based on genome-profiling approaches can be informative from a variety of perspectives, including evolutionary. As a result, the purpose of this work was to look at the genetic variation in the context of resilience against abiotic stressors in several *Rhodiola* species with different sexual systems.

## 2. Results

### 2.1. Analysis of the Genetic Diversity of Superoxide Dismutase Gene Families in Rhodiola Species

The multi-locus exon-primed intron-crossing (EPIC)-based PCR method has been widely used in plant and animal studies and is based on the design of primers selected for annealing to highly conserved exon regions [39]. EPIC-PCR has several experimental advantages, including the following: (i) primers complementary to conserved exon regions can be used over a wide taxonomic range, (ii) homologous amplified sequences can be easily identified by comparing their exon and intron regions depending on the genetic distance between taxa, and (iii) exon and intron fragments can help in the simultaneous study of genetic diversity at intraspecific and interspecific levels. The results of EPIC-PCR amplification for samples of *Rhodiola* species with primers for the SOD genes are shown in Supplemental Figure S1 (Analysis of amplification results using the EPIC-PCR technique). It was shown that the number of polymorphic amplicons was highest (63%) for *R. rosea* (KZ) and *R. semenovii* (56%) (Table 1). 

The genetic diversity of superoxide dismutase homologous genes in closely related species of phylogenetic trees indicated the lowest number of effective locus alleles in *R. semenovii* (Ne) (1042), while this index varied from 1185 in *R. linearifolia* to 1234 in *R. rosea*. *R. semenovii* had the lowest genetic diversity for all common characteristics, including the number (%) of polymorphic loci in SOD introns. *R. quadrifida* (45%) and *R. rosea* (42%) have the highest polymorphic SOD gene introns. Genetic diversity within and between *Rhodiola* species was assessed using analysis of molecular variance (AMOVA) (Table 2). The value PhiPT = 0.611 indicates that *Rhodiola* species differ significantly in SOD gene polymorphism, which can be utilized to construct genetic markers in the future. The level of intraspecific variability (61%) was found to be higher in the experiment than the level of interspecific variability (39%). Binary matrices were tested for neutrality (Tajima’s Neutrality Test) to determine the degree of genetic difference between various *Rhodiola* species in terms of SOD genes, which demonstrated divergence between the analyzed *Rhodiola* species. (Table 3). This test aims to evaluate the distribution of allele frequencies of the investigated genes across different taxonomic groups. In this context, we specifically investigated the interspecies polymorphism of the SOD genes [40,41,42]. A high (>0) D value implies a paucity of unusual alleles, indicating that the genetic polymorphism of the SOD genes is preserved without the development of any new forms.

The principal coordinates analysis (PCoA) genotype distribution approach was also applied to analyze the similarities of different *Rhodiola* species regarding SOD genes. The results are shown in Supplemental Figure S2. The allelic variations of the SOD genes are most comparable in the species *R. quadrifida*, *R. Linearifolia*, and *R. algida*, according to PCoA analysis. *R. rosea* and *R. semenovii* have been separated from other *Rhodiola* species.

### 2.2. Analysis of the Genetic Diversity of Auxin Response Factor Gene Families for Rhodiola Species

EPIC-PCR results for *Rhodiola* species revealed that use of a pair of primers (5176–5177) is the most efficient for sample differentiation. For each species of *Rhodiola*, a unique EPIC-PC profile for this gene family was obtained. EPIC-PCR analysis of amplification patterns indicated that the least variable genes of the ARF family were discovered in *R. rosea*, which contained 30% polymorphic amplicons (Supplemental Figure S3). Except for *R. algida*, this proportion was the same for the other species (54%). *R. algida* amplification spectra included up to 64% polymorphic amplicons (Table 1). 

The predicted size of EPIC-PCR fragments generated with primers for the auxin response factor gene families was 200 to 3000 bp (Figure S3).

*R. algida* also has the highest levels of genetic diversity and heterozygosity. At the same time, only *R. algida* was discovered to have a smaller number of effective Ne alleles (1396) than the total number of Na alleles (1442). It was found that the frequency of occurrence of single-allele variations surpasses the average value for the whole sample of patterns only in *R. algida* and *R. linearifolia*.

By deconstructing the trait dispersion into components of different levels, the examination of the molecular dispersion of ARF genes reflected the structure of the *Rhodiola* species (Table 2). The analysis of molecular scatter revealed that the experiment’s intraspecific variability (51%) is nearly equivalent to the interspecific variability (49%). The results show that the diverse species of *Rhodiola* sp. respond similarly to potential stress stimuli. The tests for neutrality of the different *Rhodiola* species revealed statistically significant values based on the results of the ARF gene polymorphism analysis (Table 3).

PCoA analysis was performed to identify similarities and differences between *Rhodiola* species (Figure S2B). The study discovered genetic variations in the auxin response factor gene family allelic variants in *Rhodiola* sp. The first axis (38%) separated *R. linearifolia* and *R. semenovii* from other species, whereas the second axis (28%) separated *R. algida* from *R. quadrifida* and *R. rosea*. The dioecious *R. quadrifida* and *R. rosea* were genetically most closely related, according to an examination of their positions in the system of principal coordinates. In terms of allelic variations of the auxin response factor gene family, *R. algida* varies greatly from other *Rhodiola* species.

### 2.3. Inter-Primer Binding Site (iPBS) Genome Fingerprinting Analysis for Rhodiola Species

The PBS primers used in the investigation were preliminarily evaluated to determine whether they could be utilized for genome fingerprinting and efficiently detect genetic variability among the samples analyzed. PBS primers that generated PCR fragments in small numbers were excluded from further studies. Among the tested PBS primers (2221, 2228, 2230, and 2240), the amplification profiles of the former were found to be especially beneficial in comparison to the others. These PBS primers produced homogeneous amplicon intensity profiles in adequate amounts for all the *Rhodiola* species specimens that were examined [32,36,43,44]. In total, 25 PBS primers were used to analyze the polymorphism of *Rhodiola* species specimens. The following stage involved using these PBS primers to genome fingerprint specimens of *Rhodiola* species (Supplemental Figure S4). 

The iPBS-profiling PCR approach is effective in revealing intraspecific variability within specimens of the same species. As a result, it can serve as a valuable tool for species identification in interspecific analyses. Unique and common amplicons in iPBS profiles distinctive to each species might serve as species identifiers. Some conserved amplicons may be shared by closely related species. Polymorphic bands within each species cannot be used to determine the species. A broad examination of each species using iPBS profiling, on the other hand, allows for the determination of how close specific species are to the existence of common “species” amplicons. The analysis of the amplification data revealed that the number of amplified fragments that constituted the genetic profile of the samples was unique to each species. The genetic profiles produced for various *Rhodiola* species comprised both unique and common amplicons indicative of each *Rhodiola* species (Table 1).

*R. algida* and *R. rosea* have the lowest average rates of polymorphic amplicons, with 13% and 22%, respectively. The mean unbiased heterozygosity (*uHe*) for *R. algida*, *R. rosea*, and *R. semenovii* is substantially lower than the experiment average, indicating a heterozygote deficit in these species. This might be attributed to the phenomenon of inbreeding. 

Based on the results of genetic analyses of populations derived from iPBS profiling data, it is possible to conclude that about 26% of the genetic diversity is attributable to variations between *Rhodiola* species data and 74% is related to intrapopulation variability (Table 2).

The genetic difference (*PhiPT*) between the analyzed species is *PhiPT* = 0.743 (*p* = 0.001), indicating considerable differences produced by evolutionary divergence and a limited effect of genetic drift.

Tajima’s neutrality test study verified the lack of uncommon alleles in the studied species (Table 3). PCoA indicated a *Rhodiola* species distribution in which *R. semenovii* and *R. algida* diverge from other species (Figure S2C). The results obtained demonstrate that there are only a few species-specific iPBS amplicons that are common to all specimens in the examined *Rhodiola* species, except for *R. algida*. Most iPBS amplicons are polymorphic at the species level and cannot be used for interspecific analysis. Thus, the species *R. algida* had the fewest species-specific iPBS amplicons identified.

## 3. Discussion

The study of molecular mechanisms of plant adaptation to abiotic stress factors is a timely topic in the context of global climate change [41]. Plants that grow in adverse or unstable climatic conditions, such as *Rhodiola* species studied in our work in high mountains, have accumulated unique combinations of latent variability as well as allelic variations of genes that affect the ability to tolerate environmental stress factors with which they are met during the growth season. The species’ adaptive traits have repeatedly evolved under strong selection pressure [45,46]. It is well understood that intraspecific genetic variability is the most important factor that ensures both the survival of species under adverse environmental conditions and the exploitation of their reproductive potential, allowing them to successfully resist natural selection pressures at the population level, as it is an indicator of a species’ potential ability to expand its range [47]. Species studied exhibit a high percentage of intrapopulation polymorphism compared to others, making them an important source of genetic diversity.

The mechanism of action of proteins encoding superoxide dismutase genes comprises the successive reduction and oxidation of the metal of the active center of the enzyme by superoxide anion radicals, avoiding the conversion of the superoxide anion radical into a harmful hydroxyl radical and therefore having a favorable effect on plant growth and development and their response to abiotic stress [42,48,49]. At the same time, the regulatory regions, intron sequences, and coding portions of the SOD genes had the most variability. A complicated polymorphism was discovered in the intron region of beta-amylase genes, presenting itself as prolonged insertions and deletions that can be identified by EPIC-PCR profiling [39].

The high amount of genetic polymorphism reported in this study implies the presence of several allelic forms of the SOD genes, which may be connected with the ability to adjust to unfavorable environmental circumstances. Based on the results, it can be expected that genetic variation of the SOD loci can contribute to the increase in the adaptive capacity of plants of the examined species without the creation of new forms. It can also be assumed that the examined *Rhodiola* species lacked uncommon alleles in the SOD gene families at the time of the experiment, which could lead to a dramatic decrease in their numbers.

We observed that the genetic polymorphism of the SOD genes is conserved without the creation of new morphophysiological adaptations or the production of new forms, but rather by enhancing the adaptive capacity of existing populations. This might potentially point to a recent fall in population size, implying that only genotypes descended from forms that survived an unfavorable time remained. Furthermore, because the impact of the geographical factors on the distribution of the examined species in the coordinate system was not noticed, their distribution may be related to both physiological traits and sexuality systems.

Auxin, like all the major phytohormones found in higher plants, functions as a mediator for cellular activity transmission, coordinating numerous signal transduction pathways during a stress response and regulating the effect of both external and internal stimuli [50,51]. Genes involved in diverse auxin-associated pathways express differentially under different environmental pressures, indicating the importance of auxin under various types of abiotic stresses [22,23]. ARF genes may be the components that impart specificity to the auxin response via target gene selection as transcription factors [24]. ARF gene promoters include a substantial number of cis-acting regions related to abiotic stress, indicating that ARF genes are engaged in stress protection [52,53]. Presumably, the ARF family of genes can provide plants from *Rhodiola* species with the ability to adapt to adverse climatic conditions such as a substantial temperature amplitude during the day and excessive solar radiation [22]. According to our results, there is a significant amount of genetic variation in ARF genes in studied *Rhodiola* species representatives. In contrast to the examination of polymorphism in SOD genes [48], it can be stated in this situation that the appearance of novel polymorphic forms within populations ensures the successful existence of populations, which explains the relatively high level of the Shannon index (*I*), as well as the effective *Ne* alleles number.

Both SOD family genes and ARF genes had a great variety of EPIC-PCR amplicons in all *Rhodiola* species studied. This indicates the latent diversity linked with adaptation processes that exist in all the images studied for *Rhodiola* species, allowing them to survive under demanding conditions. It may be inferred that the analyzed populations of samples for *Rhodiola* species, like related succulent plants, respond mainly similarly to stress. The high intraspecific variability values for the examined gene families might be attributed to species’ geographic isolation, which is common in many natural species, including those living in mountain environments.

Most plants’ dioecious reproduction mechanisms permit them to avoid the harmful effects of inbreeding, but under stressful situations (for example, in highlands where pollinating insects are few), their reproductive capacity can be considerably diminished [53]. Numerous studies suggest that the development of sexual differentiation under the impact of stressful situations is primarily due to conflict between the functions of selecting the reproductive strategy and the plant organism’s defensive measures under conditions of limited resources [18,19,54]. The development of species based on syngenesis is a potential adaptive approach for maintaining individual viability under adverse situations.

The activation of mobile elements and endogenous viruses, particularly LTR retrotransposons, is a critical factor in the creation of latent variability and the origin of genetic polymorphism linked with various sections of the genome [26,55]. Endogenous viruses and LTR retrotransposons play a unique role in the instability and evolution of the genome, as well as the body’s adaptability to aberrant situations [55]. They make up a considerable portion of the genome, include cis-regulatory regions comparable to protective transcription factors, and can regulate nearby genes by adjusting their expression in response to stress [56]. Some LTR retrotransposons are probably deployed directly into the genes that are most susceptible to environmental changes as a result of evolutionary adaptations [57].

In general, our data revealed significant variations in latent genomic variability for iPBS profiles amongst *Rhodiola* species, possibly due to evolutionary distinctiveness and the modest effect of genetic drift. At the same time, intrapopulation variability accounted for 74% of genetic variation.

The results of the iPBS profiling analysis for the studied samples of *Rhodiola* species revealed the level of genetic differentiation of the studied species’ populations and demonstrated the lowest average number of alleles per locus in *R. algida* and *R. rosea*, demonstrating for the same species low values of all indicators of genetic diversity. At the same time, the values of *Ne*, a marker of a species’ genetic variety, surpassed these indicators in all species, suggesting that these plants possess a reservoir of latent variability in the noncoding region of the genome, allowing it to respond to stresses that produce major genetic changes throughout evolution. According to the results of the iPBS study, the hermaphrodite species *R. semenovii* and *R. algida* are placed independently from other species. In the future, it will undoubtedly be important to examine the control of mobile elements and endogenous viruses, as well as their impact on the adaptive genome of succulent plants, including representatives of *Rhodiola* species. These succulent plants are one-of-a-kind not only phenotypically but also genetically, and they serve as distinctive representatives of the plant world for acquiring better knowledge of plant adaptation to and tolerance of abiotic conditions.

## 4. Materials and Methods

### 4.1. Plant Material

The following individual specimens from five species of the family were used for analysis *Rhodiola*: *R. rosea* L. (populations from Kazakhstan (KZ) and Russia (RU)), *R. semenovii* (Regel and Herder) Boriss., *R. linearifolia* Boriss., *R. algida* (Ledeb.) Fisch. and C.A. Mey., and *R. quadrifida* (Pallas) Fischer and Meyer. Plant samples were taken from natural populations of nature reserves in various mountainous regions of Kazakhstan; the geographical coordinates of the selection and sources of selection are presented in Table 4.

Each analyzed group of plants consisted of 10 plants (3–5 shoots from each) of the same age growing up to 5 m apart to ensure the reliability of the data obtained and minimal damage to the living plants.

Plant material was identified in the Republican State Enterprise at the Institute of Botany and Phytointroduction of the Ministry of Education and Science of the Republic of Kazakhstan.

Genomic DNA was extracted from fresh plant leaves using modified acid CTAB extraction buffer (2% CTAB, 2 M NaCl, 10 mM Na_3_EDTA, 100 mM HEPES, pH 5.3) with RNAse A treatment [58].

### 4.2. Exon-Primed Intron-Crossing (EPIC-PCR) Profiling for the Analysis of Genetic Polymorphism of Superoxide Dismutase (SOD) and Auxin Response Factor Genes for Rhodiola Species

PCR primer pairs were designed using the complete nucleotide sequences of the superoxide dismutase (SOD) and ARF6 (auxin response factor 6) family genes, which were obtained from Genbank NCBI (http://www.ncbi.nlm.nih.gov/nuccore/) (accessed on 1 February 2023) and Ensemble Plants (http://plants.ensembl.org) (accessed on 1 February 2023). The Multain software was used to perform multiple alignments of nucleotide sequences. Using the FastPCR software [59,60], “universal” EPIC primers for gene sequences in the superoxide dismutase and auxin response factor families were generated based on these data. Macrogen (Seoul, Republic of Korea) produced the primers (Table 5, Table 6 and Table 7).

EPIC-PCR reactions were performed in a 25 µL reaction mixture. Each reaction mixture contained 50 ng of template DNA, 1 × Phire^®^ Hot Start II PCR buffer with 1.5 mM MgCl_2_, 0.5 µM of each primer, 0.2 mM of each dNTP, and 0.5 µL Phire^®^ Hot Start II DNA polymerase (2U/µL) (Thermo Fisher Scientific Inc., Waltham, MA, United States). PCR amplification was carried out in a Bio-Rad Thermal Cycler T100 under the following conditions: initial denaturation step at 98 °C for 1 min, followed by 30 amplifications at 98 °C for 5 s, at 60 °C for 30 s, and 72 °C for 60 s, followed by a final extension of 72 °C for 3 min.

PCR products were separated by electrophoresis at 70V for 8 h in 1.5% agarose gel (RESOLute Wide Range, BIOzym) with 1 × TBE buffer. A Thermo Scientific (100–10,000 base pairs) GeneRuler DNA Ladder Mix (#SM0332) was used as a standard DNA ladder. The PCR products were visualized with a PharosFX Plus Imaging System (Bio-Rad Laboratories Inc., Hercules, CA, USA) with a resolution of 50 µm, after staining with ethidium bromide.

The extent of the amplicons was determined through the Quantity One program. Only clear scorable bands were used for studying genetic variability. Each band of a unique size was assumed to correspond to a unique locus. To construct a binary matrix, reproducible fragments were scored as present (1) or absent (0). Only well-detectable amplicons (polymorphic and monomorphic) were used to determine polymorphism. Amplicons whose presence/absence could not be easily differentiated were excluded from the study. The proportion of polymorphic amplicons out of the total number of amplicons for each primer was used to calculate the amount of detectable polymorphism.

GenAlex 6.5 [61] was used to calculate the total number of alleles (Na), the number of effective alleles per locus (Ne), observed heterozygosity (Ho), unexpected heterozygosity (uHE), Shannon’s information index (I), genetic differentiation index (PhiPT) among populations, and the number of unique alleles per population. Analysis of molecular variance (AMOVA) among and within populations was also calculated with GenAlex 6.5 [61].

### 4.3. Inter-Primer Binding Site (iPBS) Genome Fingerprinting

Universal PBS primers were used to evaluate the genetic variability of *Rhodiola* species specimens (Table 7). The PBS primers were 18 nucleotides long [36].

iPBS PCR reactions were performed in a 25 µL reaction mixture [32,44]. Each reaction mixture contained 50 ng of template DNA, 1 × Phire^®^ Hot Start II PCR buffer with 1.5 mM MgCl_2_, 1 µM PBS primer, 0.2 mM of each dNTP, and 0.2 µL Phire^®^ Hot Start II DNA polymerase (2U/µL) (Thermo Fisher Scientific Inc., Waltham, MA, United States). PCR amplification was carried out in a Bio-Rad Thermal Cycler T100 under the following conditions: initial denaturation step at 98 °C for 1 min, followed by 30 amplifications at 98 °C for 5 s, at 50–57 °C (depending on primer sequence) for 60 s, and at 72 °C for 60 s, followed by a final extension of 72 °C for 3 min. All PCRs were repeated at least twice for each isolate.

PCR products were separated by electrophoresis at 70V for 8 h in 1.2% agarose gel with 1 × TBE buffer. PBS primers generated in the PCR yielded distinct amplification products, showing considerable variability among the isolates belonging to different *Rhodiola* species.

## 5. Conclusions

The results obtained in this study show a high level of molecular genetic polymorphism in the coding part of the genome in the studied samples for species from the *Rhodiola* family, based on using EPIC-PCR profiling in the analysis of allelic variants of the SOD and ARF gene families. Additionally, iPBS profiling of the specimens was performed to analyze the hidden genetic variation in the noncoding part of the genomes. The high diversity in allelic variants of the SOD and ARF gene families, as well as in iPBS genome profiling, may be associated with the hidden genetic variation in the studied natural samples for species from the *Rhodiola* family and their high adaptive ability in response to adverse environmental factors, as well as with different adaptation strategies of studied species, evolutionarily determined by the diversity of reproductive systems.

## Figures and Tables

**Table 1:** Genetic diversity of *Rhodiola* species using genetic polymorphism data obtained by EPIC-PCR and iPBS profiling.

Species	Na	Ne	I	He	uHe	NPB
according to SOD genes						
*R.quadrifida*	1029	1194	0.194	0.123	0.129	44.93%
*R.rosea*	1043	1234	0.210	0.139	0.146	42.03%
*R. linearifolia*	0.884	1185	0.175	0.114	0.120	37.68%
*R. semenovii*	0.478	1042	0.043	0.026	0.028	11.59%
*R. algida*	0.768	1217	0.182	0.123	0.130	33.33%
Total	0.841	1174	0.161	0.105	0.110	33.91%
according to ARF genes						
*R.quadrifida*	1200	1295	0.264	0.174	0.183	53.68%
*R.rosea*	0.737	1201	0.168	0.114	0.120	29.47%
*R. linearifolia*	1200	1320	0.276	0.185	0.195	53.68%
*R. semenovii*	1189	1289	0.257	0.169	0.178	53.68%
*R. algida*	1442	1396	0.347	0.233	0.245	64.21%
Total	1154	1300	0.262	0.175	0.184	50.95%
according to iPBS profiling						
*R.quadrifida*	1056	1166	0.178	0.111	0.117	41.57%
*R.rosea*	0.787	1097	0.094	0.060	0.063	22.47%
*R. linearifolia*	0.921	1210	0.189	0.124	0.131	39.33%
*R. semenovii*	0.809	1155	0.137	0.090	0.095	28.09%
*R. algida*	0.674	1086	0.076	0.051	0.054	13.48%
Total	0.849	1143	0.135	0.087	0.092	28.99%

Note: Na—number of alleles; Ne—number of effective alleles per locus; I—Shannon’s information index; He—expected heterozygosity; uHE—unbiased expected heterozygosity; NPB—number (%) of polymorphic loci.

**Table 2:** Analysis of molecular variance of *Rhodiola species*.

Variability	df	SS	MS	Est. Var.	%	PhiPT	P (Rand >= Data)
according to SOD genes							
Between populations	4	295,040	73,760	6934	61%	0.611	0.001>
Within populations	45	198,900	4420	4420	39%		
Total	49	493,940		11,354	100%		
according to ARF genes							
Between populations	4	431,080	107,770	9743	49%	0.485	0.001
Within populations	45	465,200	10,338	10,338	51%		
Total	49	896,280		20,081	100%		
according to iPBS profiling							
Between populations	4	616,120	154,030	14,887	74%	0.743	0.001
Within populations	45	232,000	5156	5156	26%		
Total	49	848,120		20,043	100%		

Note: Df—number of degrees of freedom; SS—sum of squares; MS—mean square; Est. Var—variance; PhiPT—index of genetic differentiation of populations.

**Table 3:** Tajima’s neutrality test analysis.

S	ps	Θ	π	D
according to SOD genes				
88	0.926316	0.206804	0.310135	1.768874
according to ARF genes				
95	1.000000	0.223254	0.335931	1.791244
according to iPBS profiling				
87	0.977528	0.218237	0.388957	2.768256

Note: S—number of amplicons; ps—frequencies of the alleles; Θ—number of silent polymorphisms per amplicon; π—amplicons diversity; D—estimated value of Tajima’s test.

**Table 4:** Plant material used in this study.

Species	Plant Sampling Coordinates	Place of Plant Sampling
*R. rosea (KZ)*	N 50°18′34″ E 83°52′03″	Western Altai
*R. semenovii* (Regel and Herder) Boriss.	N 43°04′21″ E 76°59′07″	Trans-Ili Alatau
*R. linearifolia* Boriss.	N 43°03′29″ E 76°58′17″	Trans-Ili Alatau
*R. algida* (Ledeb.) Fisch. and C.A. Mey.	N 50°32′37″ E 76°88′94″	Western Altai
*R. quadrifida* (Pallas) Fischer and Meyer	N 50°32′83″ E 83°89′39″	Western Altai

**Table 5:** Universal primers used in the analysis of polymorphisms of allelic variations of superoxide dismutase (SOD) gene families using multi-locus EPIC-PCR profiling.

ID	Sequence (5′–3′)	Tm (°C) *	CG (%)	LC (%)	Primer Location at JX398977
5069	CGGAGGCTCTCCAAGGTCGTSTCC	73.3	66.7	81	113 → 136
5070	CCAAGGTCGTSTCCTACTACGGCCTCAC	73.6	60.7	85	123 → 150
5071	CCTCACCACTCCGCCGTACAAA	69.6	59.1	78	145 → 166
5073	AAGCCTCSGCGCGCATCATGCGTA	75.9	62.5	83	720 ← 743
5075	CTCCCACAAGTCTAGGCTGATGATTGG	68.9	51.9	93	605 ← 631

* Tm, melting temperature, calculated with 0.5 µM concentration and with 1.5 mM Mg^2+^. CG (%): the GC content of a primer is the percentage of nucleotides that are either G or C nucleotides. LC (%): linguistic sequence complexity (LC) is a measure of the ‘vocabulary richness’ of a genetic text in primer sequences.

**Table 6:** Universal primers used in the analysis of polymorphisms of allelic variations of auxin response factor gene families using multi-locus EPIC-PCR profiling.

ID	Sequence (5′–3′)	Tm (°C) *	CG (%)	LC (%)	Primer Location at XM_011009313
5175	AYTTYCCACARGGYCACAGTGARCA	69.3	50.0	81	1065 → 1089
5176	TCACAYTGGCGNTCAGTNAAGGTT	67.6	47.9	98	1922 → 1945
5177	GTCATCTGNGCRTANACTTCATCNGTCTC	68.1	48.3	91	1199 ← 1227
5178	GAAGATRTGYCTRAAYTTCCATTCRTTAYCATG	65.1	36.4	88	1451 ← 1483
5179	GTCAACAAATACAAGCTGCCAGCCTGATCT	70.8	46.7	81	3353 ← 3382

* Tm, melting temperature, calculated with 0.5 µM concentration and with 1.5 mM Mg^2+^.

**Table 7:** PBS primers used in the analyses of genetic polymorphism of *Rhodiola* species.

ID	Sequence (5′–3′)	Tm (°C) *	CG (%)	Ta (°C)
2221	ACCTAGCTCACGATGCCA	64.9	55.6	56.9
2224	ATCCTGGCAATGGAACCA	63.0	50.0	55.4
2228	CATTGGCTCTTGATACCA	57.8	44.4	54.0
2230	TCTAGGCGTCTGATACCA	60.6	50.0	52.9
2232	AGAGAGGCTCGGATACCA	63.4	55.6	55.4
2237	CCCCTACCTGGCGTGCCA	71.9	72.2	55.0
2238	ACCTAGCTCATGATGCCA	62.0	50.0	56.0
2240	AACCTGGCTCAGATGCCA	65.6	55.6	55.0
2241	ACCTAGCTCATCATGCCA	62.0	50.0	55.0
2373	GAACTTGCTCCGATGCCA	64.3	55.6	51.0

* Tm, melting temperature, calculated with 1 µM concentration and with 1.5 mM Mg^2+^; Ta, the optimal annealing temperature.

## Data Availability

Not applicable.

## Supplementary Materials

The following supporting information can be downloaded at: https://www.mdpi.com/article/10.3390/genes14040794/s1, Figure S1: Electrophoretic pattern of EPIC-PCR for *Rhodiola* species using pairs of primers to conserved regions of genes SOD: (A) pair (5069-5073); Figure S2: A plot of the distribution of Rhodiola species in the Principal Coordinates Analysis (PCoA) system; Figure S3: Electrophoretic pattern of EPIC-PCR for Rhodiola species using different pairs of primers to Auxin Response Factors; Figure S4: Electrophoretic pattern of iPBS profiling for Rhodiola species using single PBS primer.
