# Supplementary material for: Genetic Diversity in Natural Populations of Rhodiola Species of Different Adaptation Strategies
Source: Genes (Basel). 2023 Mar 25;14(4):794. doi: 10.3390/genes14040794 (PMC10137911; doi:10.3390/genes14040794)
Supplement: Supplementary file 1 [file genes-14-00794-s001.zip › genes-2266938-supplementary.pdf]

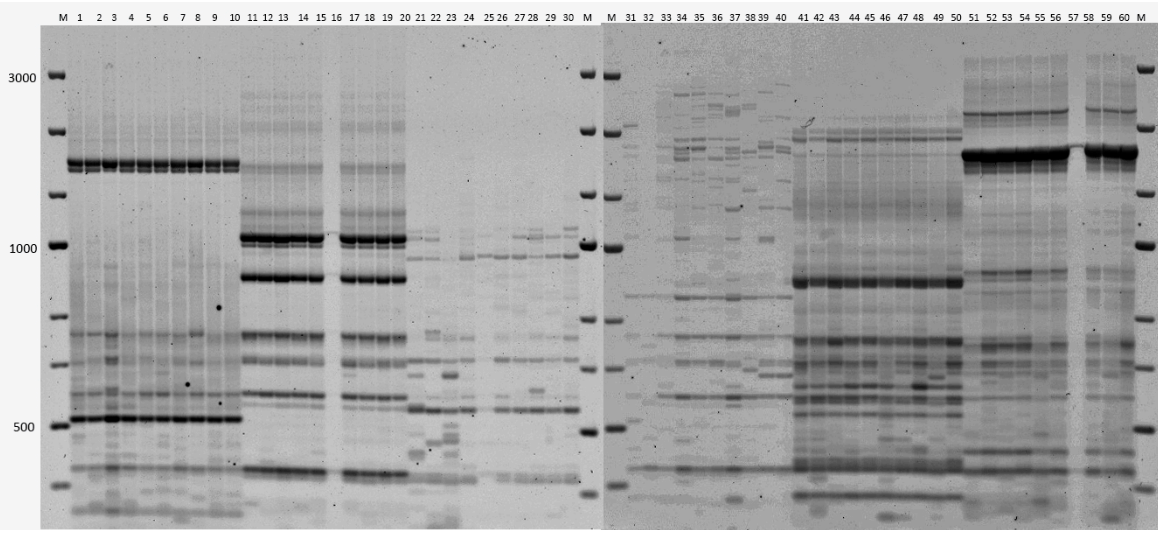
M 1 2 3 4 5 6 7 8 9 10 11 12 13 14 15 16 17 18 19 20 21 22 23 24 25 26 27 28 29 30 M
M 31 32 33 34 35 36 37 38 39 40 41 42 43 44 45 46 47 48 49 50 51 52 53 54 55 56 57 58 59 60 M
3000
1000
500

(**A**)

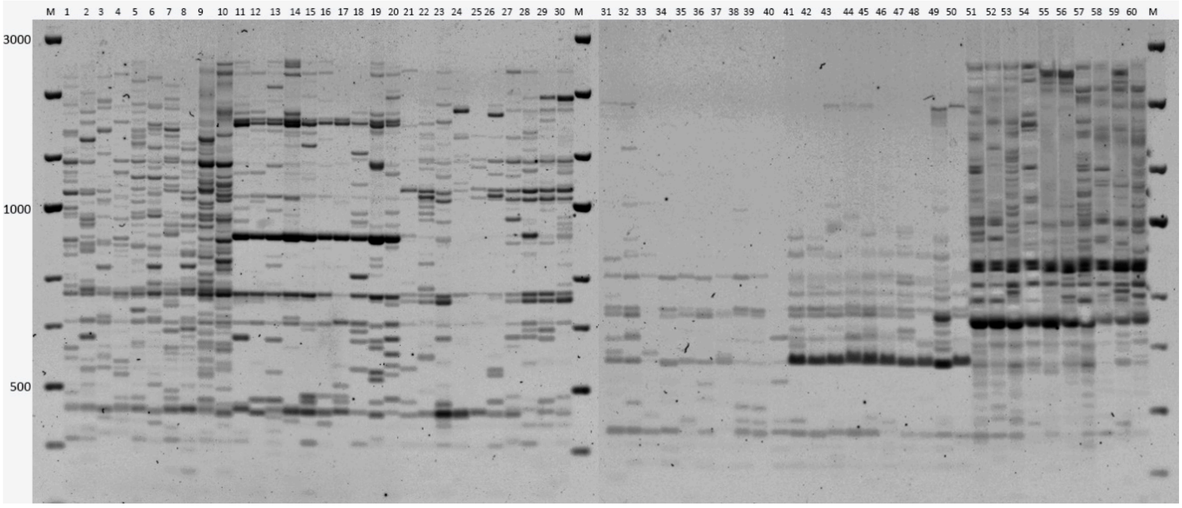
M 1 2 3 4 5 6 7 8 9 10 11 12 13 14 15 16 17 18 19 20 21 22 23 24 25 26 27 28 29 30 M
31 32 33 34 35 36 37 38 39 40 41 42 43 44 45 46 47 48 49 50 51 52 53 54 55 56 57 58 59 60 M
3000
1000
500

(**B**)

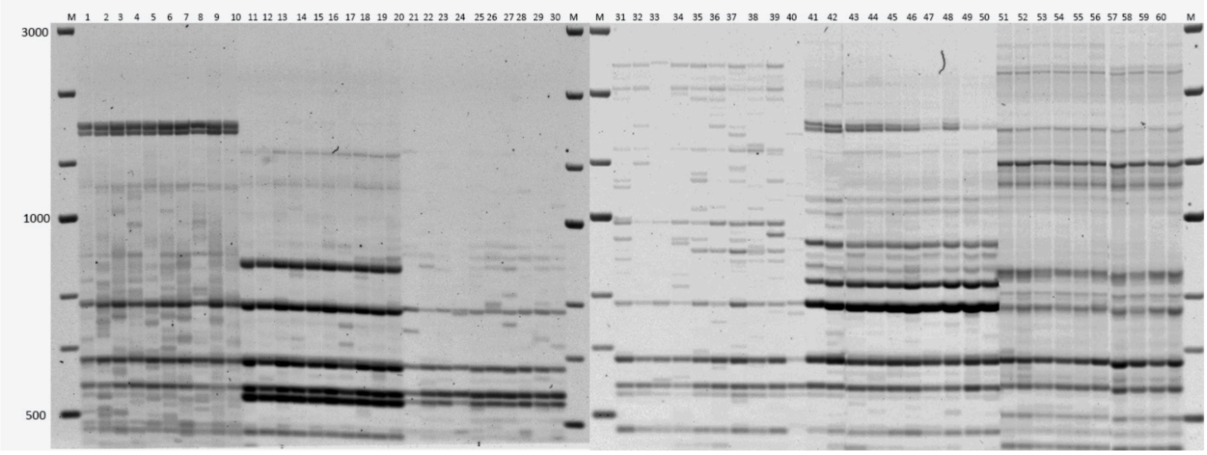
M 1 2 3 4 5 6 7 8 9 10 11 12 13 14 15 16 17 18 19 20 21 22 23 24 25 26 27 28 29 30 M
M 31 32 33 34 35 36 37 38 39 40 41 42 43 44 45 46 47 48 49 50 51 52 53 54 55 56 57 58 59 60 M
3000
1000
500

(**C**)

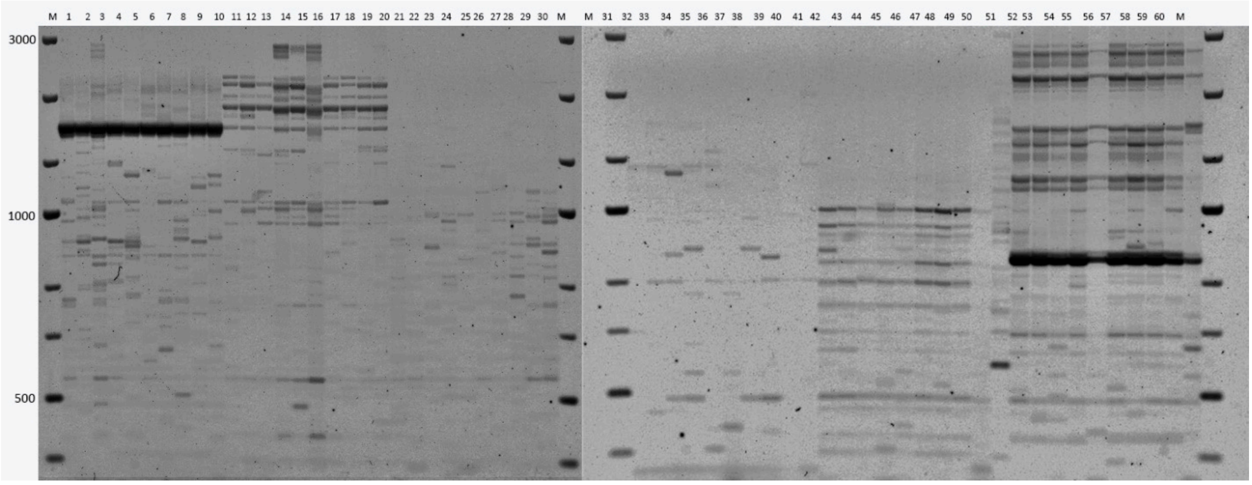

(**D**)

Figure S1. Electrophoretic pattern of EPIC-PCR for *Rhodiola* species using pairs of primers to conserved regions of genes SOD: (**A**) pair (5069-5073):): 1-10: *R. quadrifida*; 11-20: *R. rosea (KZ)*; 21-30: *R. rosea (RU)*; 31-40: *R. linearifolia*; 41-50: *R. semenovii*; 51-60: *R. algida*; (**B**) pair (5069-5075): 1-10: *R. quadrifida*; 11-20: *R. rosea (KZ)*; 21-30: *R. rosea (RU)*; 31-40: *R. linearifolia*; 41-50: *R. semenovii*; 51-60: *R. algida*; (**C**) pair (5070-5073): 25-26: *R. rosea (RU)*; 27-28: *R. semenovii*; 29-30: *R. rosea (KZ)*; 31-32: *R. quadrifida*; 33-34: *R. linearifolia*; 35-36: *R. algida*; (**D**) pair (5071-5073): 35-36: *R. rosea* (RU); 37-38: *R. semenovii*; 39-40: *R. rosea* (KZ); 41-42: *R. quadrifida*; 43-44: *R. linearifolia*; 45-46: *R. algida*. M - Thermo Scientific GeneRuler DNA Ladder Mix (100-10,000 bp).

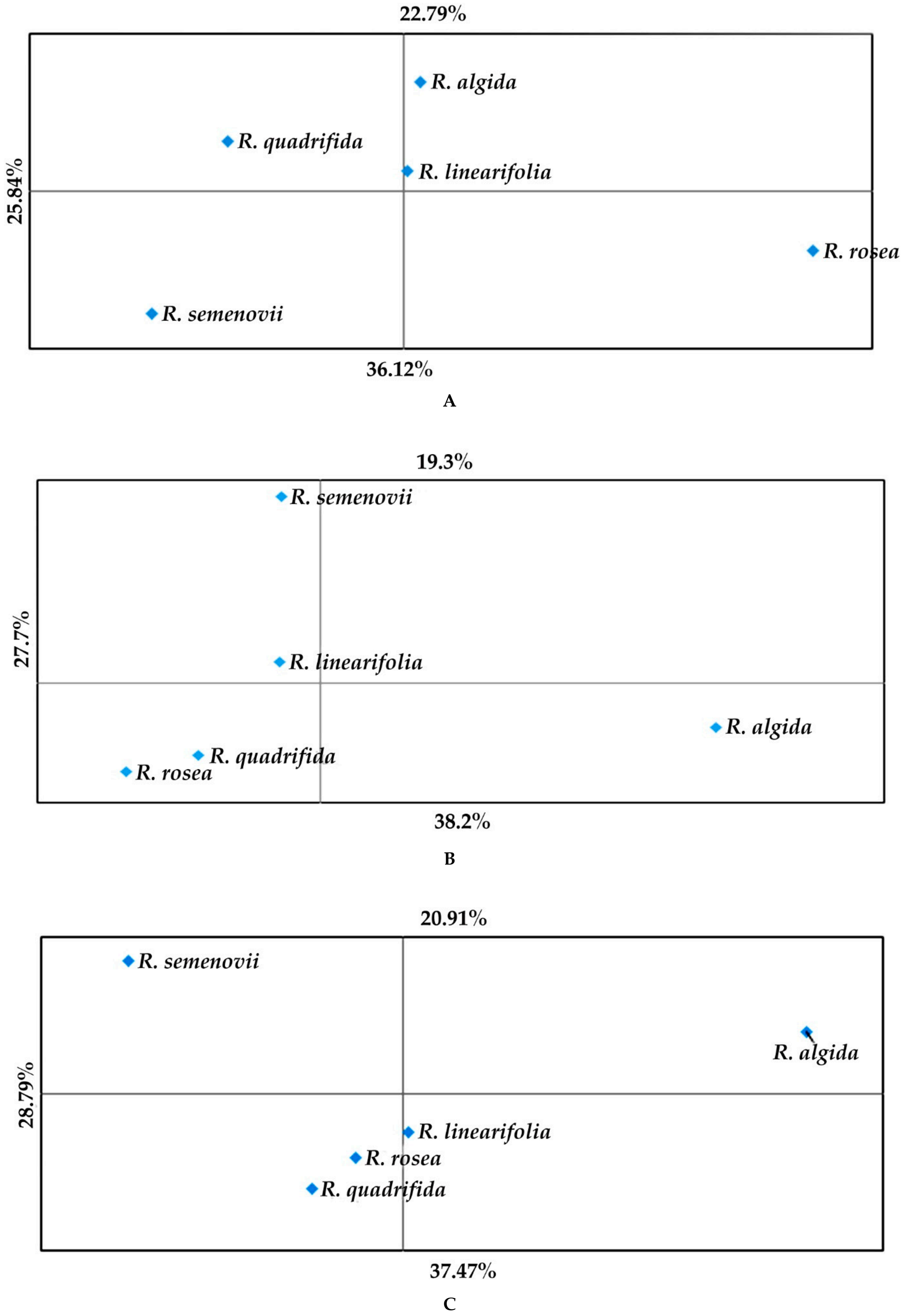

Figure S2. A plot of the distribution of *Rhodiola* species in the Principal Coordinates Analysis (PCoA) system: (**A**) by Superoxide Dismutase gene family; (**B**) by Auxin Response Factors gene family sequences examined; (**C**) by iPBS profiling results.

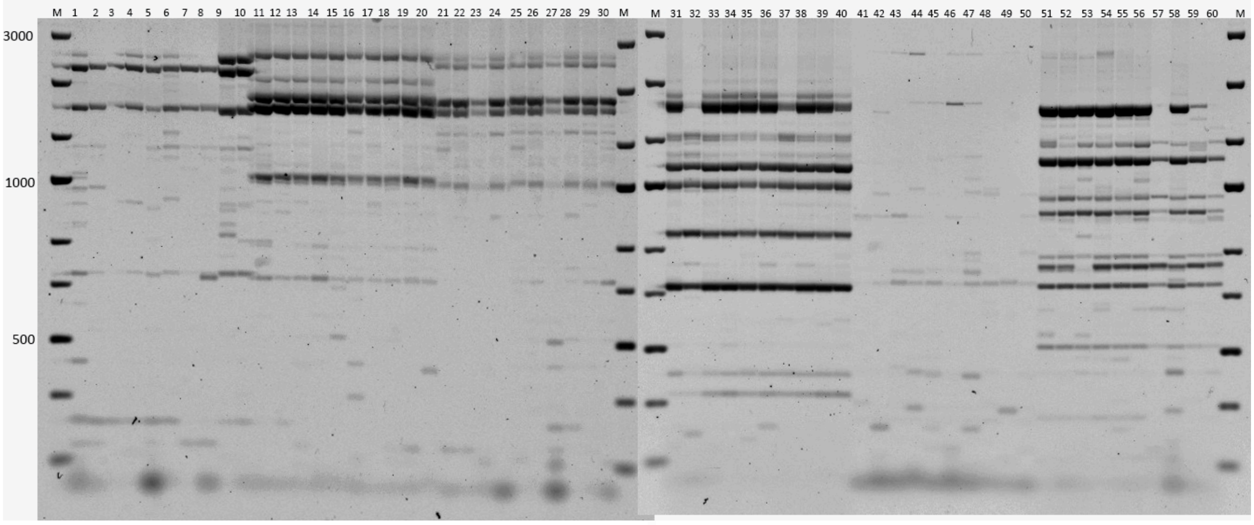

(**A**)

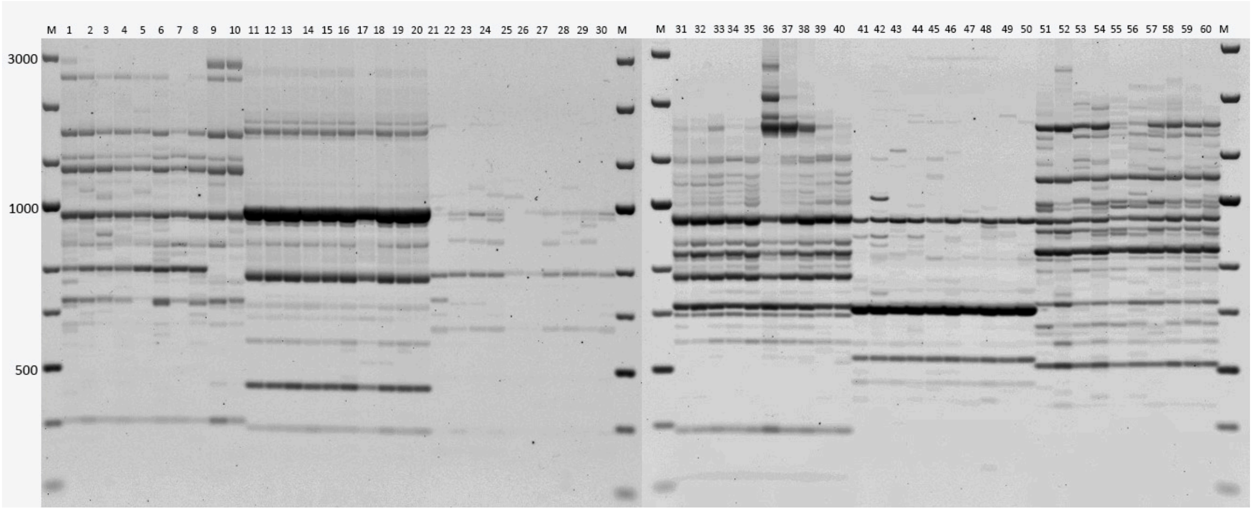

(**B**)

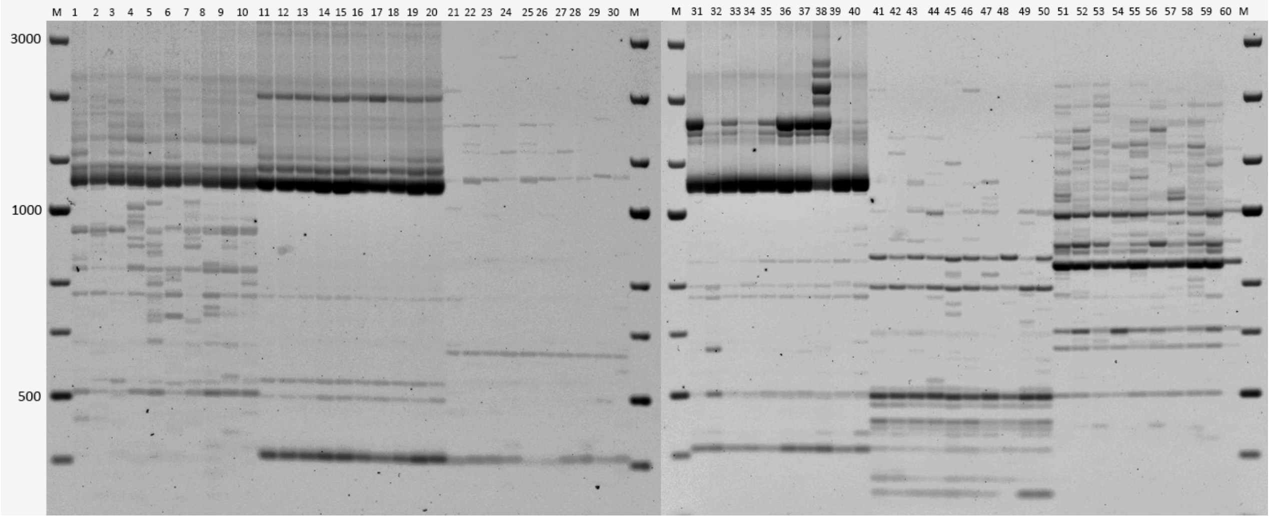

(**C**)

Figure S3. Electrophoretic pattern of EPIC-PCR for *Rhodiola* species using different pairs of primers to *Auxin Response Factors*: (**A**) pair (5176-5177): 1-10: *R. quadrifida*; 11-20: *R. rosea (KZ)*; 21-30: *R. rosea (RU)*; 31-40: *R. linearifolia*; 41-50: *R. semenovii*; 51-60: *R. algida;* (**B**) pair (5176-5179): 1-10: *R. quadrifida*; 11-20 *R. rosea (KZ)*; 21-30*R. rosea (RU)*; 31-40: *R. linearifolia*; 41-50: *R. semenovii*; 51-60: *R. algida; (*C) pair (5178-5177): 1-10: *R. quadrifida*; 11-20 *R. rosea (KZ)*; 21-30*R. rosea (RU)*; 31-40: *R. linearifolia*; 41-50: *R. semenovii*; 51-60: *R. algida*. M - Thermo Scientific GeneRuler DNA Ladder Mix (100-10,000 bp).

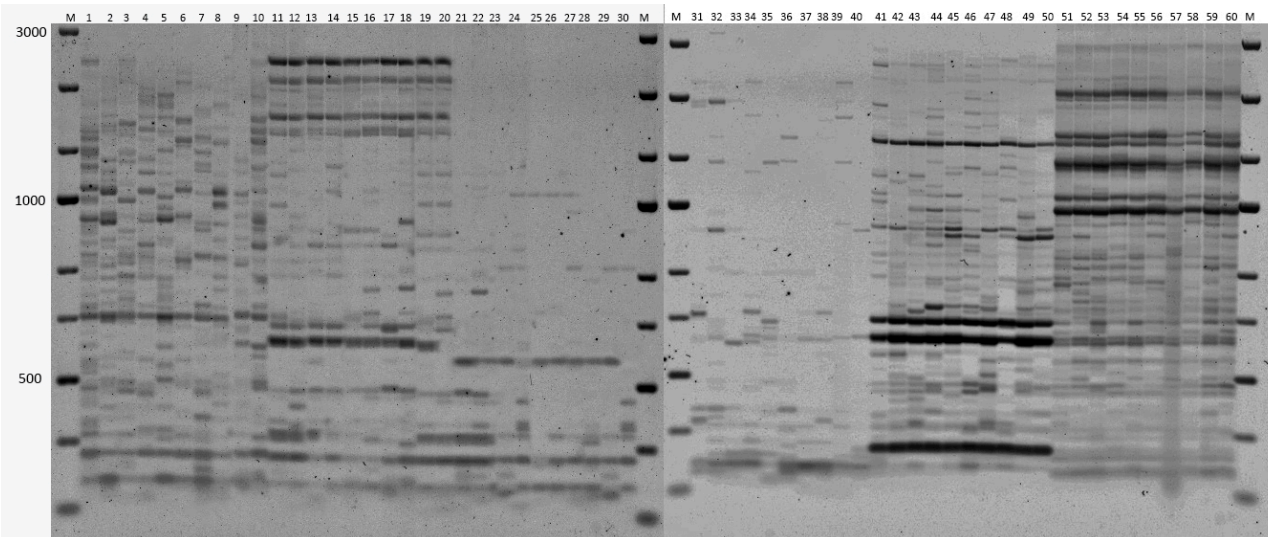
M 1 2 3 4 5 6 7 8 9 10 11 12 13 14 15 16 17 18 19 20 21 22 23 24 25 26 27 28 29 30 M
M 31 32 33 34 35 36 37 38 39 40 41 42 43 44 45 46 47 48 49 50 51 52 53 54 55 56 57 58 59 60 M
3000
1000
500

(**A**)

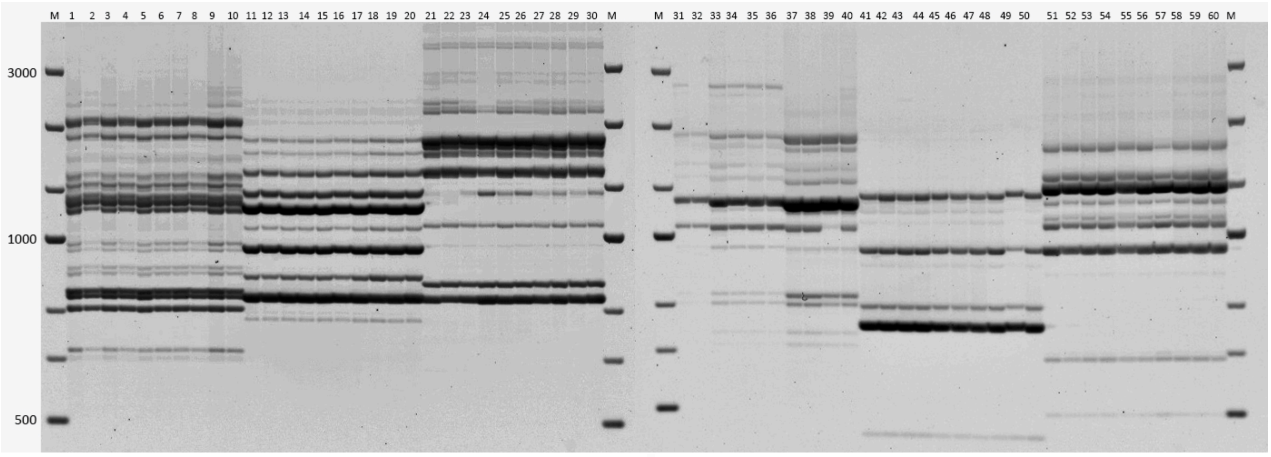
M 1 2 3 4 5 6 7 8 9 10 11 12 13 14 15 16 17 18 19 20 21 22 23 24 25 26 27 28 29 30 M
M 31 32 33 34 35 36 37 38 39 40 41 42 43 44 45 46 47 48 49 50 51 52 53 54 55 56 57 58 59 60 M
3000
1000
500

(**B**)

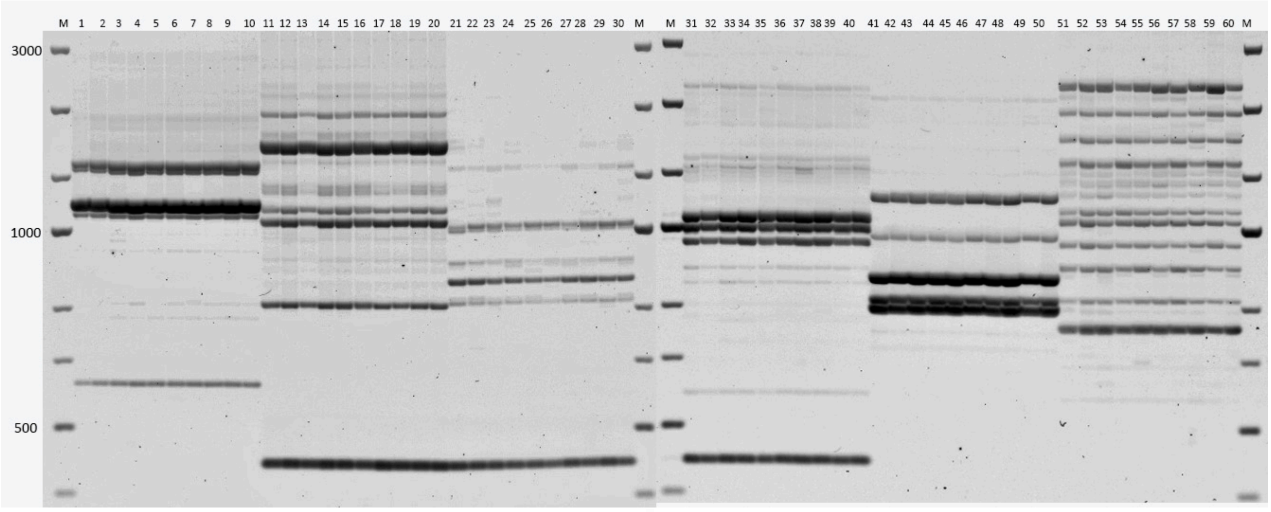
M 1 2 3 4 5 6 7 8 9 10 11 12 13 14 15 16 17 18 19 20 21 22 23 24 25 26 27 28 29 30 M
M 31 32 33 34 35 36 37 38 39 40 41 42 43 44 45 46 47 48 49 50 51 52 53 54 55 56 57 58 59 60 M
3000
1000
500

(**C**)

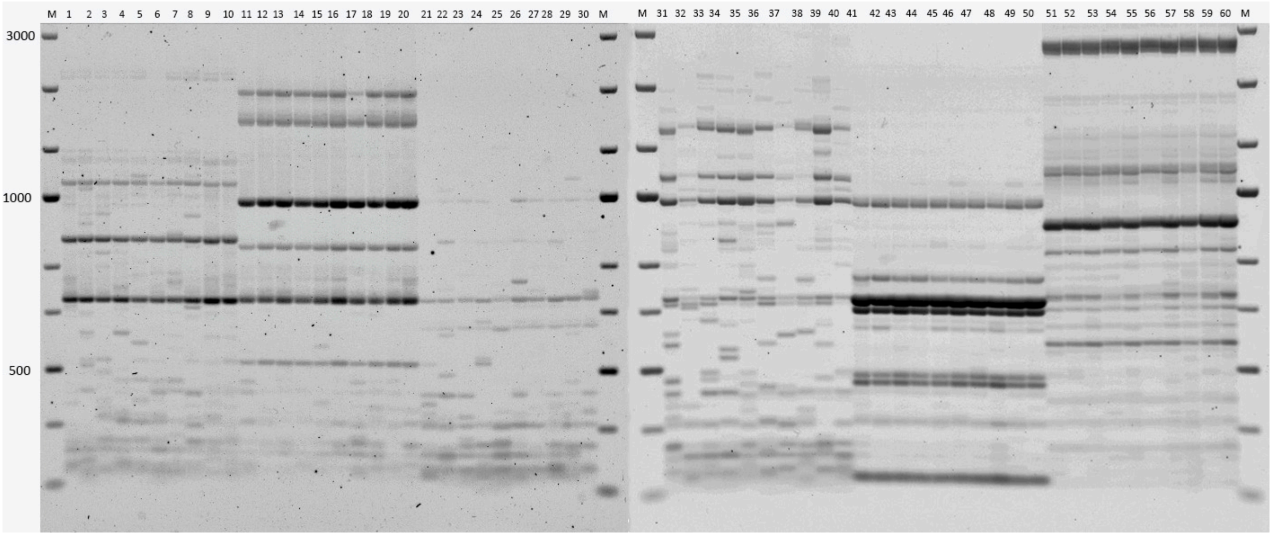

(**D**)

Figure S4. Electrophoretic pattern of iPBS profiling for *Rhodiola* species using single PBS primer: (**A**) primer 2221: 1-10: *R. quadrifida*; 11-20: *R. rosea (KZ)*; 21-30: *R. rosea (RU)*; 31-40: *R. linearifolia*; 41-50: *R. semenovii*; 51-60: *R. algida;* (**B**) primer 2230: 1-10: *R. quadrifida*; 11-20: *R. rosea (KZ)*; 21-30: *R. rosea (RU)*; 31-40: *R. linearifolia*; 41-50: *R. semenovii*; 51-60: *R. algida;* (**C**) primer 2240: 1-10: *R. quadrifida*; 11-20: *R. rosea (KZ)*; 21-30: *R. rosea (RU)*; 31-40: *R. linearifolia*; 41-50: *R. semenovii*; 51-60: *R. algida*; (**D**) primer 2228: 1-10: *R. quadrifida*; 11-20: *R. rosea (KZ)*; 21-30: *R. rosea (RU)*; 31-40: *R. linearifolia*; 41-50: *R. semenovii*; 51-60: *R. algida.*
